# Chitohexaose protects against acetaminophen-induced hepatotoxicity in mice

**DOI:** 10.1038/cddis.2016.131

**Published:** 2016-05-12

**Authors:** P K Barman, R Mukherjee, B K Prusty, S Suklabaidya, S Senapati, B Ravindran

**Affiliations:** 1 Infectious Disease Biology Group, Institute of Life Sciences, Bhubaneswar, India; 2 Translational Research Group, Institute of Life Sciences, Bhubaneswar, India; 3 Manipal University, Manipal, India

## Abstract

Acetaminophen (N-acetyl-para-aminophenol (APAP)) toxicity causes acute liver failure by inducing centrilobular hepatic damage as a consequence of mitochondrial oxidative stress. Sterile inflammation, triggered by hepatic damage, facilitates gut bacterial translocation leading to systemic inflammation; TLR4-mediated activation by LPS has been shown to have a critical role in APAP-mediated hepatotoxicity. In this study, we demonstrate significant protection mediated by chitohexaose (Chtx) in mice challenged with a lethal dose of APAP (400 mg/kg b.w.). Decreased mortality by Chtx was associated with reduced hepatic damage, increased peritoneal migration of neutrophils, decreased mRNA expression of IL-1*β* as well as inhibition of inflammasome activation in liver. Further, an alternate mouse model of co-administration of a sublethal doses of APAP (200 mg/kg b.w.) and LPS (5 mg/kg b.w.) operating synergistically and mediating complete mortality was developed. Overwhelming inflammation, characterized by increased inflammatory cytokines (TNF-*α*, IL-1*β* and so on) in liver as well as in circulation and mortality was demonstrable in this model. Also, Chtx administration mediated significant reversal of mortality in APAP+LPS co-administered mice, which was associated with reduced IL-1*β* in liver and plasma cytokines in this model. In conclusion, Chtx being a small molecular weight linear carbohydrate offers promise for clinical management of liver failure associated with APAP overdose.

Acetaminophen (N-acetyl-para-aminophenol (APAP)) toxicity remains the leading cause of acute liver failure globally, making it a major public health concern.^1, 2, 3, 4^ APAP hepatotoxicity is initiated by the production of reactive intermediate N-acetyl-p-benzoquinone imine,^5^ which under physiological conditions is detoxified by preferential binding to glutathione.^6, 7^ However, under APAP overdose, excessive induction of N-acetyl-p-benzoquinone imine results in rapid depletion of glutathione that leads to the formation of protein adducts with N-acetyl-p-benzoquinone imine causing mitochondrial oxidative stress and hepatocellular necrosis.^8^ The hepatic damage causes release of endogenous danger-associated molecular patterns (DAMPs), leading to localized sterile inflammation^9, 10^ and substantial accumulation of activated neutrophils^11^ and macrophages^12^ in liver. Sterile inflammation combined with gut bacterial translocation has been reported to trigger further activation of the innate immune system by pathogen-associated molecular patterns (PAMPs) through signaling by toll-like receptors.^13, 14, 15, 16^ Thus, overwhelming systemic inflammation along with hepatic damage causes acute liver failure to mortality.

Experimental studies with TLR4^14, 15, 16^ and mice deficient for lipopolysaccharide-binding protein^17^ have unequivocally established the role played by TLR4 and LPS in mediating APAP-induced toxicity. Successful demonstration of reduced APAP toxicity, using synthetic TLR4 antagonists^14, 18^ and inhibitor of lipopolysaccharide-binding protein,^19^ have further strengthened the TLR4-LPS nexus in APAP-induced liver dysfunction. Currently, the only available antidote for APAP toxicity is N-acetylcysteine (NAC), a ROS scavenger, which functions by replenishing glutathione and thus blocking hepatic damage.^7, 20, 21, 22^ However, NAC therapy has been found to be effective only when patients are treated at very early stages of acute overdose; delayed and prolonged administration results in enhanced toxicity and impaired liver regeneration.^23, 24, 25^

We had earlier demonstrated that chitohexose (Chtx), a low molecular weight chito-oligosaccharide, binds to TLR4 and activates macrophages by the alternate pathway (as opposed to the classical inflammatory pathway) and consequently inhibits LPS-mediated inflammation both *in vitro* and *in vivo*.^26^ We had shown that Chtx protects mice from experimental endotoxemia both prophylactically and therapeutically. A recent report demonstrating that impairment of macrophage switching from classically activated/pro-inflammatory phenotype to alternatively activated/wound repair phenotype reduces APAP toxicity, emphasizing the critical role played by alternatively activated macrophages in recovery of APAP-induced liver damage.^27^ This attracted our attention because we had shown that Chtx induces alternate activation of macrophages through its binding to TLR4. In this report, we demonstrate that Chtx protects mice from APAP-induced toxicity and mortality by reducing centrilobular hepatic damage and decreased inflammation in liver, which was further associated with increased neutrophil migration in peritoneum and inhibition of inflammasome pathway in liver. Besides, we describe that low doses of LPS and APAP act synergistically, and mutually potentiate local (liver) and systemic inflammation leading to mortality in mice, and neutralization of Chtx reverses the toxicity mutually induced by APAP and LPS in this model also.

## Results

### Chtx reduces APAP-induced mortality

**Figure 1:**
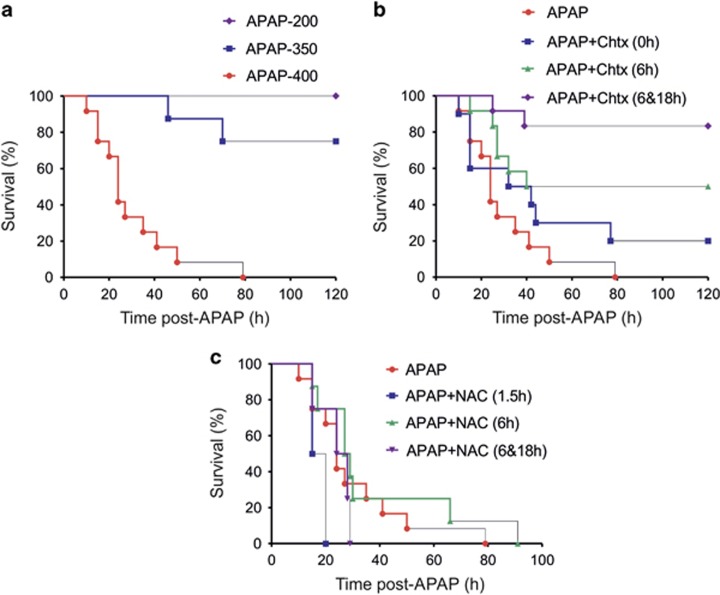
Chtx protects mice against APAP-induced mortality. (**a**) Titrated doses of APAP reveal 100% mortality only in mice receiving 400 mg/kg b.w. (APAP-200: *n*=7; APAP-350: *n*=8; APAP-400: *n*=12). (**b**) A single dose of Chtx administered at 0 or 6 h post APAP resulted in survival of 20% and 50% of mice, respectively, while two doses of Chtx injected at 6 and 18 h post APAP led to 83% survival of mice against LD100 of APAP (APAP: *n*=12, APAP+Chtx (0 h): *n*=10, APAP+Chtx (6 h): *n*=12, APAP+Chtx (6 and 18 h): *n*=12). (**c**) Administration of a single dose of NAC at 1.5 h or 6 h or two doses at 6 and 18 h failed to protect mice against LD100 of APAP (APAP: *n*=12, APAP+NAC (1.5 h): *n*=4, APAP+NAC (6h): *n*=8, APAP+NAC (6 and 18h): *n*=4)

Overnight starved male C57BL/6 mice were treated with titrated doses of APAP (200, 350 and 400 mg/kg b.w. i.p.) and followed for 5 days to score mortality. Despite showing typical signs and symptoms of APAP toxicity, mice injected with 200 mg/kg b.w. fully recovered, while 25% and 100% of animals in the 350 and 400 mg/kg b.w treatment groups died of APAP toxicity, respectively (Figure 1a). Liver necrosis, plasma inflammatory cytokines, IL-1-*β* transcript and cleaved caspase-1 in liver were significantly more in mice injected with the 400 mg dose in comparison with animals injected with 200 mg (Supplementary Figure 1). A dose of 400 mg/kg b.w. was selected for further experimentation. Mice were randomized in four treatment groups: group 1, APAP only; group 2, APAP and a single dose of Chtx injected simultaneously; group 3, APAP followed by a single dose of Chtx at 6 h post APAP and group 4, APAP followed by two doses of Chtx at 6 and 18 h post APAP for survival study. The results are shown in Figure 1b—20% and 50% of animals survived APAP-induced toxicity in group 2 and group 3, respectively, while none of the control mice (group 1) survived APAP challenge. Further, administration of two doses of Chtx at 6 and 18 h following APAP treatment significantly reduced mortality with a survival rate of 83% (group 4). NAC, on the other hand, at the dose of 300 mg/kg b.w. used in this study failed to protect mice from APAP-induced mortality, suggesting that Chtx is superior to NAC for treating APAP-induced liver toxicity (Figure 1c).

### Chtx ameliorates APAP-induced liver damage and inflammation

**Figure 2:**
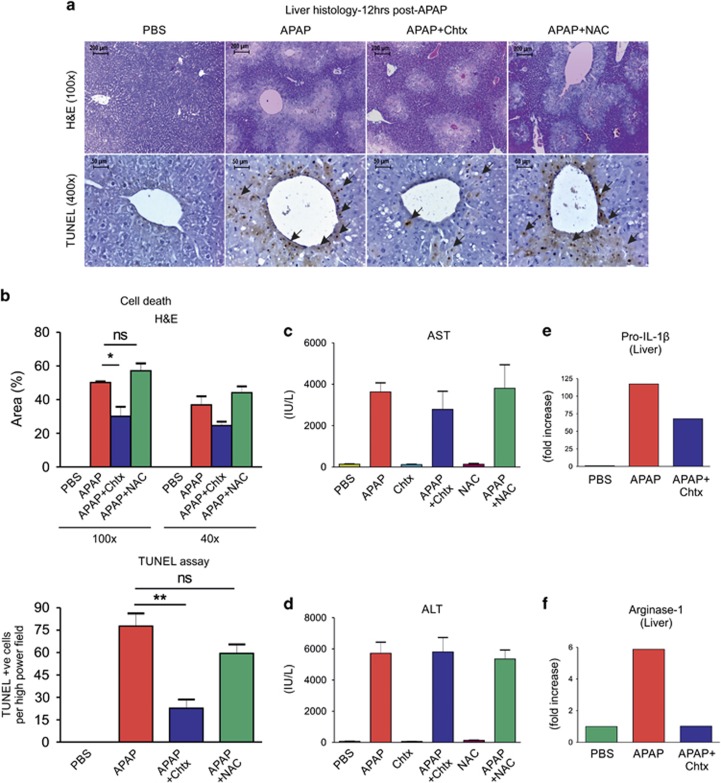
Chtx reduces hepatic necrosis and inflammation in mice challenged with APAP. (**a** and **b**) H&E staining and TUNEL assay: mice were injected with a single dose of Chtx or NAC at 6 or 1.5 h post APAP (LD100), respectively. At 12 h post APAP challenge, APAP-induced centrilobular hepatic damage was reduced upon Chtx treatment as indicated by the decreased area of necrosis and DNA fragmentation. The hepatic damage was not altered by treatment with NAC (*n*=3 mice per group, *P<0.05, **P<0.01). Necrotic area scored at × 100 and × 40 magnifications are shown. Arrow heads indicate TUNEL-positive cells. (**c** and **d**) Plasma AST and ALT levels at 12 h post APAP were comparable in mice injected with APAP or APAP+Chtx or APAP+NAC (PBS: *n*=5, APAP: *n*=3, APAP+Chtx: *n*=3, APAP+NAC: *n*=5). (**e** and **f**) Q-PCR analysis for IL-1*β* and *Arg1* expression in liver of mice injected with APAP or APAP+Chtx at 12 h post APAP challenge. Data were normalized with *β*-actin (PBS: *n*=5, APAP: *n*=6, APAP+Chtx: *n*=6, IL-1 *β* 2^−ΔcT^ values; PBS −740.4±225, APAP −26.52±10.99, APAP+Chtx −52.53±15.53, *P*=0.201 (APAP *versus* APAP+Chtx), *Arg1* 2^−ΔcT^ values; PBS 140.5±101, APAP 840±606.4, APAP+Chtx 112.7±113.1, *P*=0.265 (APAP *versus* APAP+Chtx)). Results were expressed as mean±S.E.M.

To test whether improved survival observed in Chtx treatment of APAP hepatotoxicity correlated with the degree of liver injury, histological evaluation of paraffin-sectioned livers collected at 12 h post administration of APAP was performed. Chtx administration at 6 h post APAP injection remarkably decreased centrilobular hepatic necrosis in response to APAP challenge as shown by H&E staining (APAP, 50.14±0.63% *versus* APAP+Chtx, 30.07±5.6% area of damage; *P=0.0242 at × 100 magnification; Figures 2a and b, upper panels). This was further correlated with the degree of DNA fragmentation; the number of terminal deoxynucleotidyl transferase-mediated dUTP nick-end labeling (TUNEL)-positive cells in necrotic area of liver in response to APAP was significantly decreased by treatment with Chtx (APAP, 77.73±8.5 *versus* APAP+Chtx, 22.67±5.86 number of TUNEL-positive cells per high-power field, **P=0.006; Figures 2a and b, lower panels). Despite the difference in hepatic damage as shown by histology, plasma levels of liver enzymes (alanine amino transferase and aspartate amino transferase) were comparable in both the groups at 12 h post APAP injection (Figures 2c and d). Correlating to the mortality, NAC did not alter hepatic damge as revealed by H&E staining, TUNEL assay and plasma levels of aspartate amino transferase/alanine amino transferase (Figures 2a–d). Further, to establish an association of Chtx-mediated reduction of APAP-induced hepatic damage to liver inflammation, we quantified pro-IL-1*β* transcripts in liver at 12 h post APAP administration. In comparison with the 117-fold increase in pro-IL-1 *β* expression in APAP-challenged mouse livers with respect to vehicle control, Chtx treatment resulted in only 67-fold increase in IL-1*β* message, revealing significant amelioration of APAP-induced inflammation in liver by treatment with Chtx (Figure 2e).

Arginase-1 (*Arg1*) is predominantly present in liver and initiates a catalytic reaction leading to the conversion of arginine to proline, an essential component of collagen formation during wound healing. As a consequence, plasma level of *Arg1* is increased during liver injury, making it a liver injury-specific biomarker with more sensitivity than other standard parameters.^28, 29^ The ability of Chtx in modulating the effect of APAP-induced expression of liver-specific *Arg1* was studied. As expected, APAP upregulated *Arg1* transcripts in liver and Chtx decreased this upregulated expression to basal level (Figure 2f). This observation positively correlated with our findings on reduced liver damage and mortality in Chtx-treated group of mice. However, liver arginase activity was decreased in mice treated with APAP at 12 h post treatment and came back to basal level at 24 h. Besides, although a single dose of Chtx following APAP showed liver arginase activity similar to APAP-exposure alone at 12 h post APAP, two doses of Chtx maintained reduced arginase activity at 24 h post APAP treatment (Supplementary Figure 1F).

### Migration of neutrophils and level of chemokines are increased in peritoneum in the presence of Chtx

**Figure 3:**
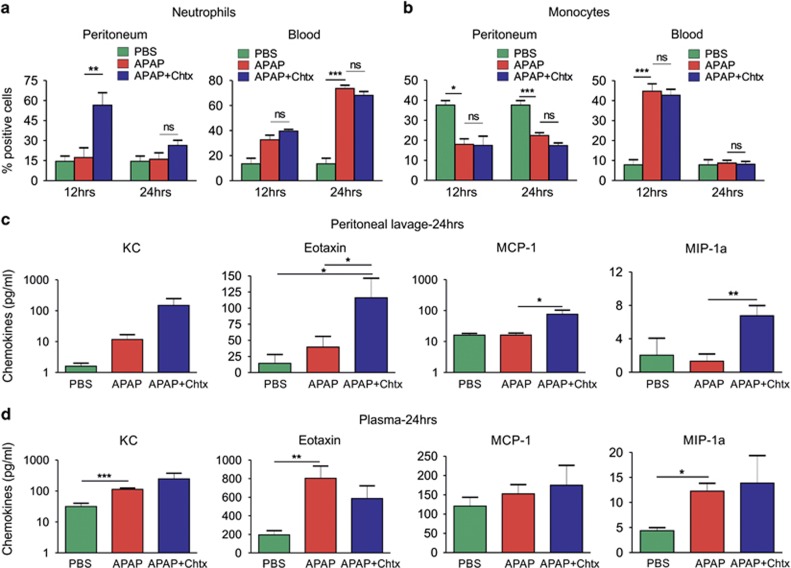
Chtx induces neutrophil migration and chemokine secretion in peritoneum of mice. (**a** and **b**) Mice were injected with PBS, APAP alone or APAP followed by a single dose of Chtx at 6 h post APAP or two doses of Chtx at 6 and 18 h post APAP. Monocytes (CD14+Ly6G−) and neutrophils (CD14-Ly6G+) from peritoneum and blood at 12 or 24 h post APAP treatment were analyzed by flow cytometry. (PBS: *n*=4, APAP: *n*=6, APAP+Chtx: *n*=6, **P<0.01). (**c** and **d**) After 24 h of treatment as mentioned above, chemokines in peritoneal exudates and plasma were measured by multiplex bioassay. KC, Eotaxin, MCP-1 and MIP-1a were upregulated in peritoneum of mice treated with APAP+Chtx, whereas their levels were comparable in plasma between APAP- and APAP+Chtx-treated groups (PBS: *n*=4, APAP: *n*=7, APAP+Chtx: *n*=7, *P<0.05, **P<0.01, ***P<0.001). Results were expressed as mean±S.E.M.

Given the fact that neutrophils and monocytes/macrophages have a crucial role in inducing inflammation and wound repair during injury,^11, 12, 30, 31^ we scored these immune cells in both peritoneum and circulation at 12 and 24 h post APAP treatment by flow cytometry. No significant change was observed in Ly6G+ neutrophil percentage in peritoneum of mice challenged with APAP, while mice injected with APAP and Chtx induced significant migration of neutrophils in the peritoneum at 12 h post APAP injection (**P<0.01, Figure 3a, left). The percentage of circulating neutrophils, on the other hand, increased at 12 h in mice treated with APAP with or without Chtx and reached to significantly higher levels at 24 h post treatment (***P<0.001; Figure 3a, right). Mice injected with APAP showed significant decrease in monocytes (Ly6G-CD14+) in peritoneum at 12 h post injection (*P<0.05), which persisted till 24 h (***P<0.001) and Chtx treatment did not alter this decrease (Figure 3b, left). On the contrary, at 12 h post treatment, percentage of circulating monocytes were significantly increased (***P<0.001) in mice in both treatment groups and returned back to basal levels at 24 h (Figure 3b, right).

Quantification of KC, eotaxin, MCP-1 and MIP-1a in peritoneal lavage and plasma of mice at 24 h post treatment with APAP was undertaken with a view to study the influence of Chtx on APAP-mediated regulation of chemokine secretion. Chtx administration in APAP-treated mice significantly elevated peritoneal levels of the chemokines, revealing development of increased chemokine gradient in peritoneum in response to Chtx treatment (Figure 3c). Assessment of plasma chemokines revealed a significant increase in the levels of KC, eotaxin and MIP-1a by APAP. This increase in plasma levels were also observed in mice treated with APAP+Chtx and there was no quantitative difference between these two treatment groups (Figure 3d). Similarly, levels of plasma inflammatory cytokines, namely, TNF-*α*, IL-1*β*, IL-4, IL-5, IL-6 and IL-12p70 were also comparable in mice treated with APAP and APAP+Chtx (Supplementary Figure 2).

### Chtx blocks APAP-mediated activation of inflammasome pathway

**Figure 4:**
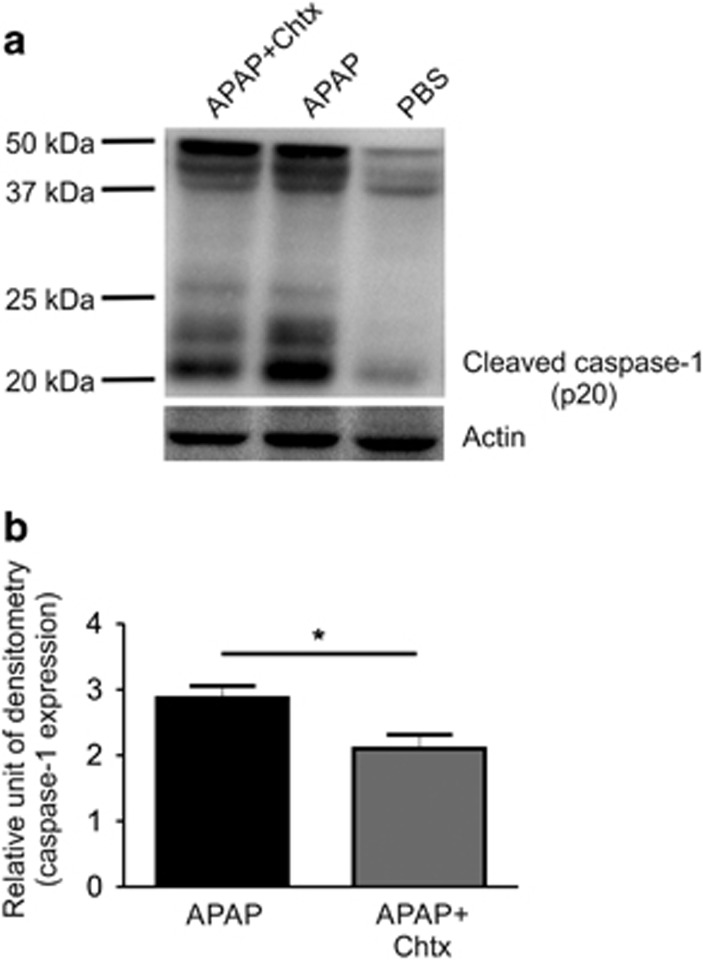
APAP-mediated activation of caspase-1 is reduced by Chtx. (**a** and **b**) Mice were injected with PBS, APAP alone or APAP followed by two doses of Chtx at 6 and 18 h post APAP. At 24 h post APAP, caspase-1 activation in liver was examined by scoring cleaved caspase-1 by western blotting (*n*=3 mice per group, *P<0.05). Actin was used as the loading control. Results were expressed as mean±S.E.M.

NLRP (NLR family, pyrin domain-containing) inflammasome, a multi-protein complex containing a member of the NLRP family, an adapter protein ASC and pro-caspase-1, is activated in response to DAMPs, released by tissue damage. The function of this complex is to convert inactive pro-caspase-1 into active caspase-1, which in turn cleaves pro-IL-1*β* and pro-IL-18 into their active forms that finally trigger the inflammatory cascade.^32, 33^ Absence of any of the components of inflammasome complex has been shown to result in reduced APAP hepatotoxicity.^13^ Further, a recent study has demonstrated that benzyl alcohol protects against APAP-mediated liver damage partly through blocking the cleavage of pro-caspase-1.^34^ The possibility of Chtx blocking APAP-induced activation of inflammasome pathway was tested by studying cleaved caspase-1 in liver extracts. Consistent with earlier findings by other investigators, a marked increase in cleaved caspase-1 was observed in liver of mice upon APAP treatment as shown by western blotting and this elevated cleaved caspase-1 was significantly decreased by Chtx treatment suggesting that Chtx significantly blocks APAP-induced activation of inflammasome pathway (*P<0.05; Figures 4a and b).

### LPS enhances susceptibility of mice towards APAP-induced mortality

**Figure 5:**
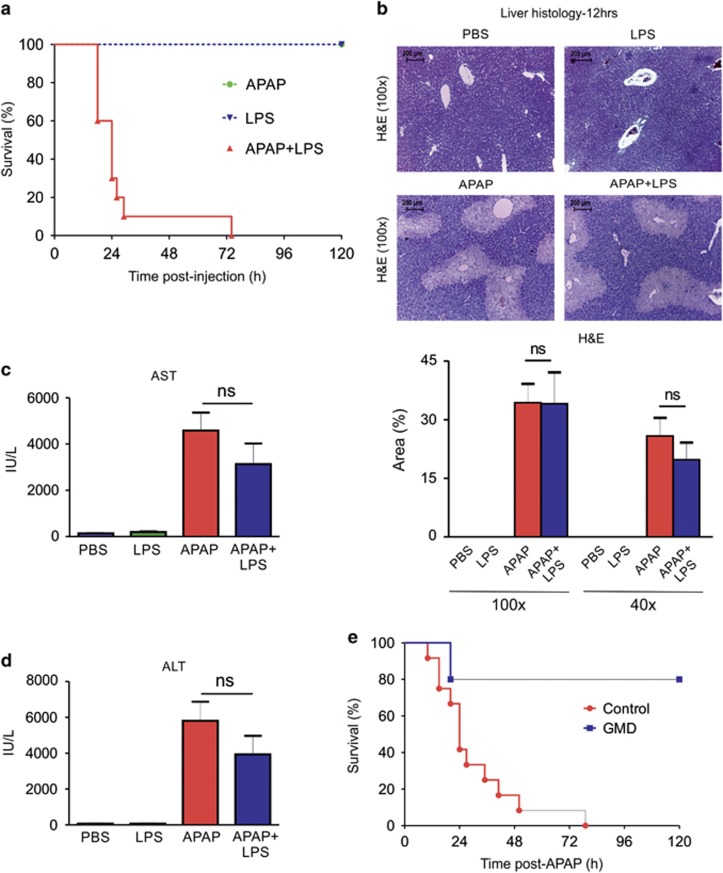
Non-lethal doses of APAP and LPS mutually induce mortality in mice. (**a**) Kaplan–Meier plot showing survival/mortality of mice, treated with APAP (200 mg/kg b.w.) alone, LPS (5 mg/kg b.w.) alone or APAP (200 mg/kg b.w.) and LPS (5 mg/kg b.w.) together (APAP: *n*=6, LPS: *n*=10, APAP+LPS: *n*=10). (**b**) H&E staining of liver biopsy (× 100 magnification) from mice 12 h after treatment with PBS, APAP, LPS and APAP+LPS showing comparable liver necrosis between APAP and APAP+LPS treatment groups. Necrotic area scored at × 100 and × 40 magnifications are shown. (**c** and **d**) Plasma AST/ALT were comparable between APAP- and APAP+LPS-treated mice at 12 h post treatment (PBS: *n*=6, APAP: *n*=6, LPS: *n*=6, APAP+LPS: *n*=7). Results were expressed as mean±S.E.M. (**e**) Survival/mortality of gut microbiota-depleted (GMD) and age-matched control C57BL/6 mice, treated with APAP (400 mg/kg b.w.) (GMD: *n*=5, Control: *n*=12)

The observation that LPS pre-exposure intensifies sensitivity to APAP hepatotoxicity in mice provides a direct evidence for the critical role played by LPS in APAP-induced liver failure.^35^ Further, resistance of lipopolysaccharide-binding protein KO mice to APAP-mediated liver injury and inhibition of APAP toxicity by lipopolysaccharide-binding protein inhibitory peptides demonstrated that LPS-mediated activation of the innate immune system is essential for APAP-induced liver failure.^17, 19^ We reasoned that exogenous administration of a low dose of LPS and non-lethal dose of APAP could lead to synergistic potentiation of toxicity and disease progression. This was tested by co-administration of APAP (200 mg/kg b.w.) and LPS (5 mg/kg b.w.) in mice. At this dose, neither APAP nor LPS caused mortality of mice (100% survival) while co-administration of the two resulted in 100% mortality (Figure 5a). Histological evaluation of liver sections showed no significant potentiation of APAP-induced liver necrosis by LPS administration, which was further reflected in plasma levels of aspartate amino transferase and alanine amino transferase, suggesting that LPS does not significantly increase induction of liver necrosis by low doses of APAP (Figures 5b–d). However, LPS increased both local (liver) and systemic inflammation when treated along with APAP as discussed below. On the basis of these observations, we conclude that LPS contributed by microbiota in mice are largely responsible for the observed inflammation and mortality in APAP-induced toxicity. This was experimentally tested in APAP-induced liver failure by scoring the mortality of mice in which the gut microbiota was depleted by prolonged antibiotic treatment. The results demonstrated significant protection of gut microbiota-depleted mice against APAP as compared with antibiotic untreated mice (Figure 5e).

### APAP and LPS mutually enhance liver inflammation and activate inflammasome pathway in mice

**Figure 6:**
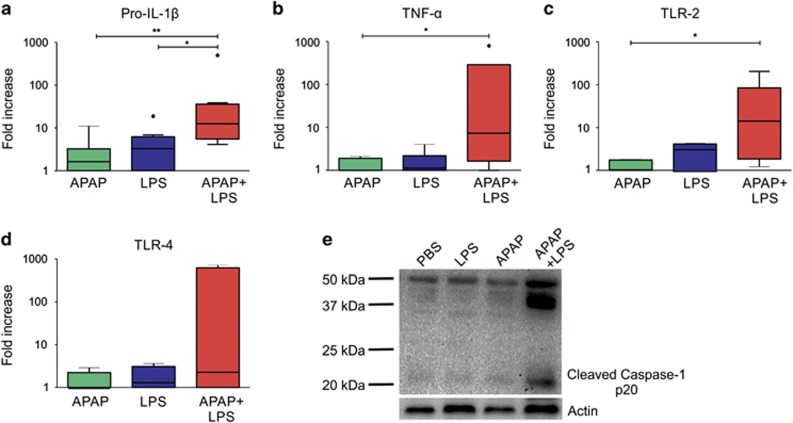
APAP and LPS mutually potentiate liver inflammation and activate caspase-1. (**a**–**d**) mRNA expression of pro-IL-1*β*, TNF-*α*, TLR2 and TLR4 in liver of mice treated with APAP, LPS and APAP+LPS measured by qPCR at 12 h post treatment. Data were presented as fold change with respect to PBS controls (*n*=6–8 mice per group, *P<0.05, **P<0.01). Results were expressed as mean±S.E.M. (**e**) Western blot shows presence of cleaved caspase-1 in livers of mice at 12 h post treatment with APAP and LPS together. Actin was used as the loading control

To test the status of inflammation in liver due to the synergistic effect of APAP and LPS, liver mRNA expression of inflammatory cytokines and TLRs were quantified at 12 h post treatment. There was no significant increase in pro-IL-1*β* and TNF-*α* transcripts in livers of mice individually treated with APAP and LPS, while co-administration of APAP and LPS resulted in log order increase in message for pro-IL-1*β* and TNF-*α* (Figures 6a and b). Similarly, expression of TLR2 (significantly) and TLR4 (non-significantly) increased in livers of mice injected with APAP and LPS together as compared with animals injected only with LPS or APAP (Figures 6c and d). Next, we examined whether APAP and LPS mutually potentiate the activation of inflammasome pathway. Non-lethal doses of APAP and LPS were unable to activate the inflammasome pathway as determined by the absence of cleaved caspase-1 in liver extracts, while co-administration of mice with APAP and LPS revealed marked increase in cleaved caspase-1, demonstrating synergistic activation of inflammasome pathway by APAP and LPS (Figure 6e).

### APAP and LPS mutually potentiate systemic inflammation in mice

**Figure 7:**
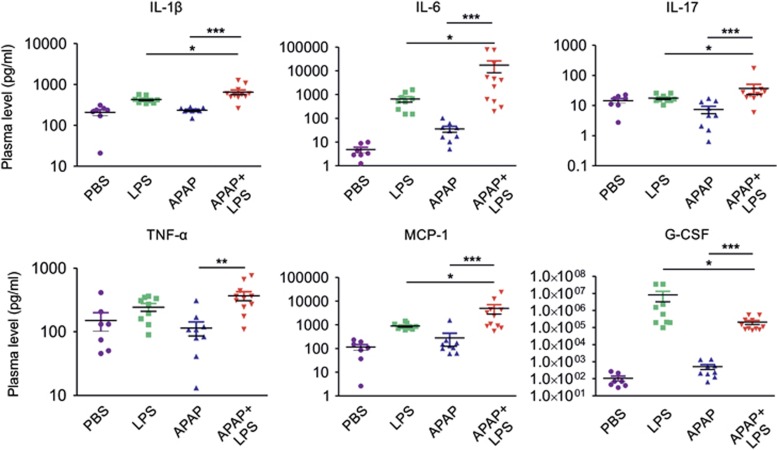
APAP and LPS mutually potentiate systemic inflammation—multiplex analysis for plasma cytokines. Mice were treated with PBS, LPS, APAP or APAP+LPS and at 12 h post treatment. Plasma cytokines were measured by multiplex bioassay. Plasma IL-1*β*, IL-6, IL-17, TNF-*α* and MCP-1 were upregulated only in mice treated with both APAP and LPS but not with APAP or LPS alone at 12 h post treatment. G-CSF was downregulated in mice treated with APAP+LPS as compared with only LPS (PBS: *n*=7, LPS: *n*=9, APAP: *n*=9, APAP+LPS: *n*=11, *P<0.05, **P<0.01, ***P<0.001). Results were expressed as mean±S.E.M.

Increased inflammation in liver of mice co-administered with APAP and LPS was associated with systemic inflammation as shown by elevated levels of plasma mediators, particularly inflammatory cytokines. Analysis by multiplex cytokine array revealed that except IL-6, there was no significant change in other cytokines in plasma of mice treated only with APAP, whereas only LPS treatment significantly increased plasma IL-1*β*, IL-6, MCP-1 and G-CSF levels in comparison with vehicle controls at 12 h post treatment. Further, co-administration of APAP and LPS significantly augmented the levels of IL-1*β*, IL-6, IL-17, TNF-*α* and MCP-1 in comparison with treatments only with APAP or LPS, indicating synergism between APAP-mediated hepatic injury and LPS-mediated innate immune activation. Unexpectedly, G-CSF level was significantly elevated in plasma of mice treated with only LPS and it decreased significantly upon treatment with both APAP and LPS as compared with only LPS (Figure 7).

### Chtx reduces mortality, liver injury and inflammation induced by treatment with APAP and LPS

**Figure 8:**
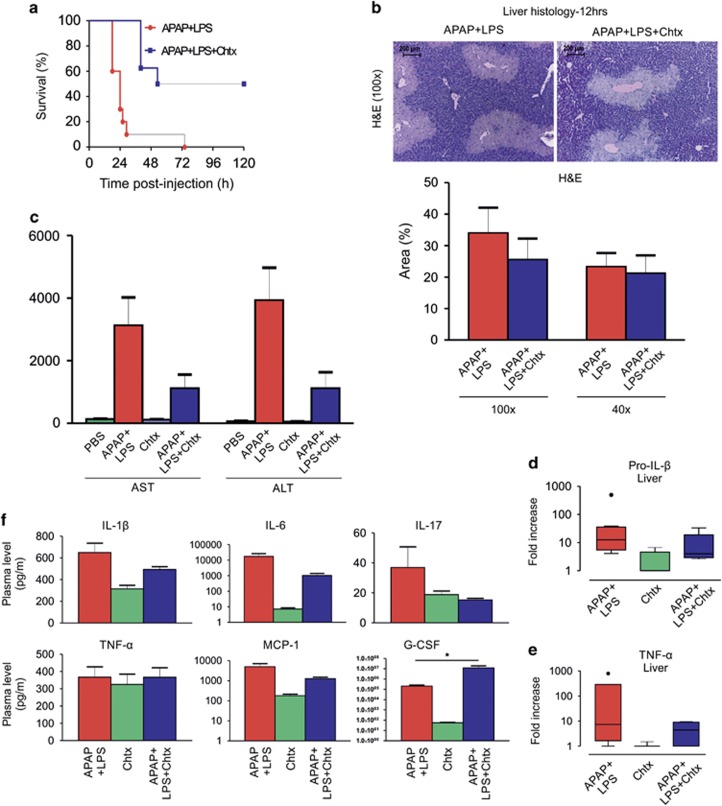
Chtx reduces mortality and hepatotoxicity induced by synergistic action of APAP and LPS in mice. (**a**) Kaplan–Meier plot showing survival/mortality of mice—Chtx offers 50% protection from APAP+LPS-induced mortality (APAP+LPS: *n*=10, APAP+LPS+Chtx: *n*=8). (**b**) H&E staining of liver biopsy (× 100 magnification) from mice 12 h after treatment with APAP+LPS and APAP+LPS+Chtx (*n*=3 mice per group). Necrotic area scored at × 100 and × 40 magnifications are shown. (**c**) Plasma AST/ALT in APAP+LPS injected mice are decreased by Chtx at 12 h post treatment (PBS: *n*=6, APAP+LPS: *n*=7, Chtx: *n*=6, APAP+LPS+Chtx: *n*=4). (**d** and **e**) qPCR analysis of IL-1*β* and TNF-*α* in liver of mice at 12 h post treatment; fold change in mRNA expression with respect to PBS controls is shown (*n*=3–8 mice in each group). (**f**) Plasma cytokines in mice at 12 h post treatment as measured by multiplex bioassay are shown. Chtx marginally decreased IL-1*β*, IL-6, IL-17 and MCP-1 and significantly increased G-CSF in response to APAP+LPS (APAP+LPS: *n*=11, Chtx: *n*=9, APAP+LPS+Chtx: *n*=6, *P<0.05). Results were expressed as mean±S.E.M.

The synergistic model of co-administration of APAP and LPS in which exacerbated local (liver) as well systemic inflammation and mortality was observed was used to test the efficacy of Chtx as a therapeutic agent. Chtx was administered in mice along with APAP and LPS and mortality was scored. Data revealed 50% reversal of APAP+LPS-induced mortality upon simultaneous treatment with Chtx. Decreased mortality was further associated with plasma levels of liver enzymes (Figures 8a and c). Area of damage and pro-IL-1*β* transcripts in liver of mice given a combined dose of APAP, LPS and Chtx were found to be decreased (non-significantly) as compared with APAP+LPS-treated ones, whereas no difference was found in liver TNF-*α* expression between these two groups of mice (Figures 8b–e). Plasma cytokine read-outs showed that Chtx marginally reduced elevated levels of IL-1*β*, IL-6, IL-17 and MCP-1 in response to APAP+LPS, without affecting TNF-*α*. On the other hand, plasma G-CSF content, which was reduced in mice combinedly treated with APAP+LPS, was significantly increased by Chtx (Figure 8f).

## Discussion

The present study highlights the critical role of bacterial endotoxin LPS and microbiota in mediating APAP-induced hepatotoxicity and it also demonstrates that a carbohydrate Chtx, an LPS antagonist, induces protection against APAP-induced liver failure in mice. The observations of this investigation further reveal that the deleterious effect of APAP can be amplified by augmented inflammation and activation of inflammasome pathway by exogenous administration of LPS. These findings offer credence to the notion that APAP essentially leads to release of DAMPs by necrosis of liver cells and that further inflammation and mortality are essentially mediated by endogenous LPS from microbiota owing to a leaky gut.^36, 37, 38^ This study also demonstrates that blockade of LPS-mediated activation by a small molecular weight carbohydrate Chtx, which activates macrophages by alternate pathway through TLR4,^26^ abrogates APAP toxicity in mice. The protection against APAP toxicity by Chtx appears to be mediated primarily by blocking the inflammasome activation pathway as shown by decreased cleaved caspase-1 in liver and also partly by reducing hepatic damage and liver tissue inflammation.

Although APAP toxicity is predominantly attributed to overdose of the drug, there are notable instances of APAP poisoning in individuals who were on therapeutic dosing.^39^ Growing evidence suggests the indispensible role of endogenous LPS and pre-existing inflammation in hepatotoxicity, mediated by xenobiotic agents.^36, 37, 38^ The finding that LPS priming renders mice more vulnerable to APAP toxicity while LPS tolerization lead to hypo-toxicity to APAP further corroborates the critical role of endogenous LPS in APAP-mediated liver dysfunction.^35, 40^ In this context, our earlier observation that Chtx blocks LPS-mediated inflammation and endotoxemia in mice^26^ suggested that Chtx could potentially block APAP toxicity also, because LPS is needed for mediating such toxicity. We demonstrate that Chtx administration significantly reduces APAP-induced mortality in mice and protective effect is enhanced when Chtx is administered at later time points or by increasing the number of doses. These observations indicate that Chtx is predominantly effective in curing APAP-induced hepatotoxicity when applied at progressive and later stages of liver injury, making it superior to NAC for management of APAP-induced acute liver damage. The probable underlying mechanism behind Chtx-mediated protection against lethal APAP dose was found to be inhibition of inflammasome pathway and partly by reduced hepatic damage as shown by histology and *Arg1* and liver inflammation as shown by decreased IL-1*β*. Hepatocellular necrosis, inflammation, both local (as shown by liver IL-*β* transcripts) and systemic (as shown by plasma cytokines), and inflammasome activation correlate with mortality in mice by lethal *versus* non-lethal dose of APAP (Supplementary Figure 1). It is thus not surprising that Chtx-mediated protection of APAP toxicity results in significant decrease of liver damage, IL-1*β* expression and cleavage of caspase-1 in liver.

The role of specific chemokines and certain immune cell subsets in liver injury, inflammation and repair are well known.^11, 12, 41, 42^ Increased chemokine secretion post injury has been shown to trigger infiltration of neutrophils, macrophages and NKT cells in the damaged area. In the present study, we found a significant increase in plasma chemokines after lethal APAP challenge and this increase was not significantly altered by co-treatment with Chtx. Interestingly, Chtx treatment in APAP-administered mice enhanced chemokine density and displayed increased influx of neutrophils in the peritoneum. The significance of peritoneal infiltration of neutrophil and improved survival of mice against APAP is currently not clear.

Although mice models of APAP toxicity have been widely used in the past, in this study, an additional lethal model of APAP toxicity was developed, in which co-administration of non-lethal doses of APAP and LPS induced 100% lethality due to synergy between a PAMP such as LPS and DAMPs generated by APAP. This model of mortality demonstrated that it could be used in studies to investigate the intrinsic role of PAMPs in liver damage. Augmented inflammation, tissue-specific (increased pro-IL-1*β* and TNF-*α* expression in liver) as well as systemic (increased plasma IL-*β*, IL-6, IL-17 and MCP-1 levels) were responsible for the observed mortality in this model (Figures 6 and 7). Further, secretion of an anti-inflammatory cytokine, such as G-CSF that gets elevated after LPS challenge as a compensatory mechanism,^43, 44^ was impaired in APAP-LPS model of toxicity developed in this study. We propose that increased plasma G-CSF is a natural anti-inflammatory mechanism to counter-balance excessive inflammation by LPS. However, in the presence of APAP-mediated liver damage, this counter-active mechanism is impaired, resulting in overwhelming inflammation as manifested by decreased G-CSF in mice injected with APAP and LPS—not surprisingly, Chtx-mediated protection in this model was associated with recovery of higher levels of G-CSF. Chtx-mediated protection of mice in the model of APAP+LPS treatment was also associated with reduced liver damage and decreased inflammation, thus demonstrating that neutralization of LPS by Chtx partially reversed the toxic effects induced by APAP and LPS. Our data showing protection of gut microbiota-depleted mice against APAP-induced mortality (Figure 5e) along with previous findings of reduced APAP toxicity in germ-free mice by others^15^ further confirm the crucial role of PAMPs secreted by gut microbiota in APAP-mediated liver failure. Together, these results indicate synergy between TLR signaling by PAMPs and sterile inflammation mediated by DAMPs in APAP-LPS-induced toxicity/mortality in mice and the ability of Chtx in blocking the liver toxicity. The current drug of choice NAC for clinical management of APAP-induced liver toxicity was not found to block mortality in the toxicity model system used in the present study (Figure 1c) although NAC was reported several years ago to be decreasing inflammation in experimental animals^21^ based on which clinical trials were undertaken and found to be useful.^22^ The results of present study indicate that Chtx is far superior to NAC in mitigating *in vivo* toxicity mediated by APAP.

Intracellular signaling from HMGB1-RAGE, ATP-P2X7 and/or mitochondrial DNA-TLR9 interactions post tissue damage has been reported to activate NLRP3 inflammasome complex, leading to the production of active IL-1*β* and IL-18 by proteolytic cleavage mediated by cleaved caspase-1.^13, 33, 34^ Thus, activation of inflammasome complex and proteolytic cleavage of pro-caspase-1 is a critical event in maintaining the inflammatory cascade, as it forms a bridge between TLR signaling and sterile inflammation. Our demonstration of NRLP activation by co-administration of non-lethal dose of APAP and low dose of LPS (either of them in isolation was insufficient to mediate toxcity/mortality) further validates our interpretation on synergy between TLR4-mediated signaling and sterile inflammation. In addition, significantly diminished activation of inflammasome by Chtx in mice challenged with lethal APAP dose further confirms the critical role of inflammasome activation in APAP-induced acute liver failure and it also indicates that blockade of this pathway can lead to protection against APAP-induced mortality.

In conclusion, the present study offers a vital model of APAP-induced hepatotoxicity in synergy with PAMPs such as LPS and reveals critical role of LPS and gut microbiota in APAP-induced liver failure. It further demonstrates that Chtx, which inhibits LPS-mediated endotoxemia by competitive binding through TLR4, protects mice against APAP-induced inflammation and mortality. Thus, Chtx being a non-immunogenic small molecular weight carbohydrate with the ability to block TLR4-mediated inflammation and to activate anti-inflammatory alternate pathway of macrophages appears to be a promising molecule for clinical management of APAP toxicity and appropriate clinical trials could lead to its use in intensive care medicine units.

## Materials and Methods

### Reagents

APAP, LPS from *Escherichia coli* serotype O55:B5 and NAC were purchased from Sigma-Aldrich (St. Louis, MO, USA). Hexa-N-acetyle Chtx was from Dextra Laboratories Ltd. (West Berkshire, UK). Bio-Plex Pro Mouse Cytokine 23-plex Assay kit was from Bio-Rad (Hercules, CA, USA). cDNA Synthesis Kit and Q-PCR SYBR mix were from Agilent Technologies (Santa Clara, CA, USA). Arginase activity assay kit was from Sigma-Aldrich (St. Louis, MO, USA). Colorimetric TUNEL assay kit was from Promega (Madison, WI, USA). Anti-mouse antibodies to CD14-(PE) and Ly6G-(perCP-Cy5.5) were from eBiosciences (San Diego, CA, USA) and BD Biosciences (New Jersey, NJ, USA) respectively. Antibodies to caspase-1 and actin were from Santacruz Biotechnology (Dallas, TX, USA) and Sigma-Aldrich, respectively.

### Animals

C57BL/6 mice were obtained from National Institute of Immunology, New Delhi, India, originally from Jackson Laboratories (Bar Harbor, Maine, USA). Breeding and maintenance were carried out at Institute of Life Sciences, Bhubaneswar, India. Male mice (8–10 weeks old) were used throughout the investigation. Experimental protocol was approved by Institutional Animal Ethics Committee (IAEC) for conducting experiments using mice and approved protocols were strictly followed. The study was carried out in strict accordance with the recommendations of the Committee for Prevention of Cruelty and Safety of Experiments with Animals (CPCSEA) that supervises care and use of laboratory experimentation through their nominees in the Institutional animal ethics committee.

### APAP-induced hepatotoxicity

APAP solution was prepared in sterile DPBS at 20 mg/ml, heated at 55 °C with agitation and used freshly. Mice were starved for 18 h and injected intraperitoneally (i.p.) with APAP at 200, 350 and/or 400 mg/kg body weight (b.w.). Mice were killed by CO_2_ inhalation at 12 or 24 h post APAP injection for sampling or observed till day 5 for mortality.

### Antibiotic treatment

C57BL/6 mice were gavage-fed with antibiotic cocktail containing vancomycin (50 mg/kg b.w.), neomycin (100 mg/kg b.w.), metronidazole (100 mg/kg b.w.), ampicillin (100 kg/kg b.w.) and amphotericin B (1 mg/kg b.w.) twice daily over a period of 21 days along with ampicillin (1 mg/ml) in drinking water *ad libitum* to generate gut microbiota-depleted mice. Microbiota estimate revealed about 97% reduction of cultivable bacteria (CFU) in stool of antibiotic-treated mice in comparison with untreated control mice.

### Chtx, NAC and LPS treatment

Solutions of Chtx, NAC and LPS were prepared in sterile DPBS and stored at −20 °C. Chtx was administered i.p. at 10 mg/kg b.w. as our earlier finding showed inhibition of LPS-induced inflammation by Chtx at a dose of 250 *μ*g per mouse (i.e.,10 mg/kg b.w. for a mouse of 25 g, which is the average body weight of healthy adult mouse).^26^ Treatment was given simultaneously or 6 h post APAP challenge once or at 6 and 18 h post APAP challenge twice. NAC was dosed at 300 mg/kg b.w. as it has been used as a standard dose in literature.^7^ Administration was performed i.p. once at 1.5 or 6 h post APAP or twice at 6 and 18 h post APAP. A single i.p. dose of LPS (10 mg/kg b.w.) given simultaneously with APAP (200 mg/kg b.w) was used for the second model of APAP toxicity used in this study.

### Sample collection and processing

Blood collected in ACD anticoagulant (15% v/v) by cardiac puncture after euthanasia were aliquoted for analysis by flow cytometry and the rest centrifuged at 3000 r.p.m. for 10 min for plasma collection. Three milliliters of chilled DPBS were injected in the peritoneum, left for 5 min and aspirated to collect peritoneal exudates. Cells were pelleted at 400 × *g* for 5 min and used immediately for flow cytometry analysis. Cell-free peritoneal lavage and plasma samples were stored at −80 °C for cytokine assay. Liver tissues were collected in Trizol, RIPA buffer or 10% formalin for RNA isolation, protein extraction or histology, respectively.

### Measurement of liver enzymes and arginase activity

Plasma levels of aspartate amino transferase and alanine amino transferase were measured using clinical chemistry analyzer (ERBA Chem-5X, Transasia, Mumbai, India). Arginase activity in liver tissue extracts was measured using Arginase activity assay kit following the manufacturer's instructions (Sigma-Aldrich, St. Louis, MO, USA).

### Histopathology

Formalin-fixed liver tissues were embedded in paraffin and cut into 5*-μ*m thick sections. Replicate sections were stained either with H&E or TUNEL for microscopic assessment of centrilobular necrosis and DNA fragmentation, respectively. Area of necrosis was scored for whole tissue sections and the numbers of TUNEL-positive cells were counted in 10 high-power fields (× 400 magnification) using ImageJ software.

### Q-polymerase chain reaction

Total RNA was isolated from liver samples using Trizol (Life Technologies, Santa Clara, CA, USA) and first strand cDNA was prepared using a kit following the manufacturer's instructions. Real-time Q-PCR was performed, cT values were normalized with *β*-actin and results were expressed as fold change with respect to PBS control. Primers used in the study are shown in Table 1.

### Cytokine assay

Cytokine levels in plasma and peritoneal exudates were measured using Bio-plex Pro Assays-23-plex following the manufacturer's instruction and data were acquired in Bio-Plex 200 system (Bio-Rad).

### Flow cytometry analysis

Whole blood (50 *μ*l) and peritoneal lavage cells were stained with anti-mouse antibodies to CD14-(PE) and Ly6G-(perCP-Cy5.5) following a standard protocol. Stained cells were acquired and analyzed in BD LSRFortessa, using BD FACS Diva software (version 7.0).

### Western blot analysis

Protein was extracted from liver tissues using RIPA buffer and western blot analysis for caspase-1 was performed according to standard protocols. Densitometric analysis of protein bands was performed using ImageJ software. Actin was used as sample loading control.

### Statistics

Kaplan–Meier plots and statistical analysis were performed using GraphPad Prism software (version 5.01) and results were expressed as mean±S.E.M. Groups were compared by two-tailed non-parametric *t*-test and *P* values less than 0.05 were considered as significant (at 95% confidence intervals).

## Figures and Tables

**Table 1:** Primers used in the study

Primer name	Sequence
IL-1*β*	F-5′-TGCCACCTTTTGAGAGTGATG-3′ R-5′-AAGGTCCACGGGAAAGACAC-3′
TNF-*α*	F-5′-AGGCACTCCCCCAAAAGATG-3′ R-5′-TTTGCTACGACGTGGGCTAC-3′
Arginase-1	F-5′-TTTTAGGGTTACGGCCGGTG-3′ R-5′-CCTCGAGGCTGTCCTTTTGA-3′
TLR2	F-5′-CGTTGTTCCCTGTGTTGCTG-3′ R-5′-CAGAGCTGGCGTCTCCATAG-3′
TLR4	F-5′-AATCCCTGCATAGAGGTAGTTCC-3′ R-5′-GGTGGTGTAAGCCATGCCA-3′
*β*-Actin	F-5′-CTAGGCACCAGGGTGTGATG-3′ R-5′-TGGCCTTAGGGTTCAGGGG-3′

## Abbreviations

APAP: acetaminophen
DAMPs: danger-associated molecular pattern
TLRs: toll-like receptors
Chtx: Chitohexaose
LPS: lipopolysaccharide
TNF-*α*: tumor necrosis factor-*α*
IL: interleukin
NAC: N-acetylcysteine
