# Supplementary material for: Chitohexaose protects against acetaminophen-induced hepatotoxicity in mice
Source: Cell Death Dis. 2016 May 12;7(5):e2224–. doi: 10.1038/cddis.2016.131 (PMC4917664; doi:10.1038/cddis.2016.131)
Supplement: Supplementary Figure Legends [file cddis2016131x1.docx]

**Supplementary Figure 1.** Mice were injected with APAP at 200 (non-lethal dose) or 400mg/kg b.w. (lethal dose) or volume matched PBS and following parameters were measured at 12hrs post-injection. (A) H&E staining of paraffin sectioned livers showed remarkably higher hepatic damage in mice treated with lethal dose of APAP as compared to non-lethal dose. Necrotic area scored at 100x and 40x magnifications are shown. (B) Plasma AST/ALT showing no difference between groups of mice treated with lethal and non lethal doses of APAP (n=3 to 6 mice per group). (C) Multiplex bioassay showed significantly higher levels of inflammatory cyrokines in plasma of mice treated with lethal dose of APAP as compared to non-lethal dose (PBS: n=12, APAP-200: n=6, APAP-400: n=6, *p<0.05, ***p,0.001). Results were expressed as mean±SEM. (D) Caspase-1 activation in liver of mice was examined by scoring cleaved caspase-1 by Western blot. Actin was used as loading control. (E) q-PCR showed many fold higher expression of pro-IL-1β in liver of mice treated with lethal dose of APAP as compared to non-lethal dose (n= 6 mice per group). (F) Mice were injected with PBS, APAP alone or APAP followed by a single dose of Chtx at 6hrs post-APAP or two doses of Chtx at 6 and 18hrs post-APAP. Arginase 1 activity was measured in liver tissue extracts at 12 and 24hrs post-APAP administration (n=3 mice per group).

**Supplementary Figure 2. Multiplex assay for cytokines.** APAP-induced systemic inflammation was not significantly altered by Chtx. Mice injected with PBS, APAP alone or APAP followed by Chtx at 6hrs post-APAP. Elevated plasma levels of TNF-α, IL-1β, IL-4, IL-5, IL-6 and IL-12p40 12hrs after administration of APAP was not significantly altered by treatment with Chtx. Mean+SEM of 6 mice per group is shown (*p<0.05, **p<0.01)**.**Results were expressed as mean±SEM.
