# Supplementary figures and images for: Chitohexaose protects against acetaminophen-induced hepatotoxicity in mice
Source: Cell Death Dis. 2016 May 12;7(5):e2224–. doi: 10.1038/cddis.2016.131 (PMC4917664; doi:10.1038/cddis.2016.131)

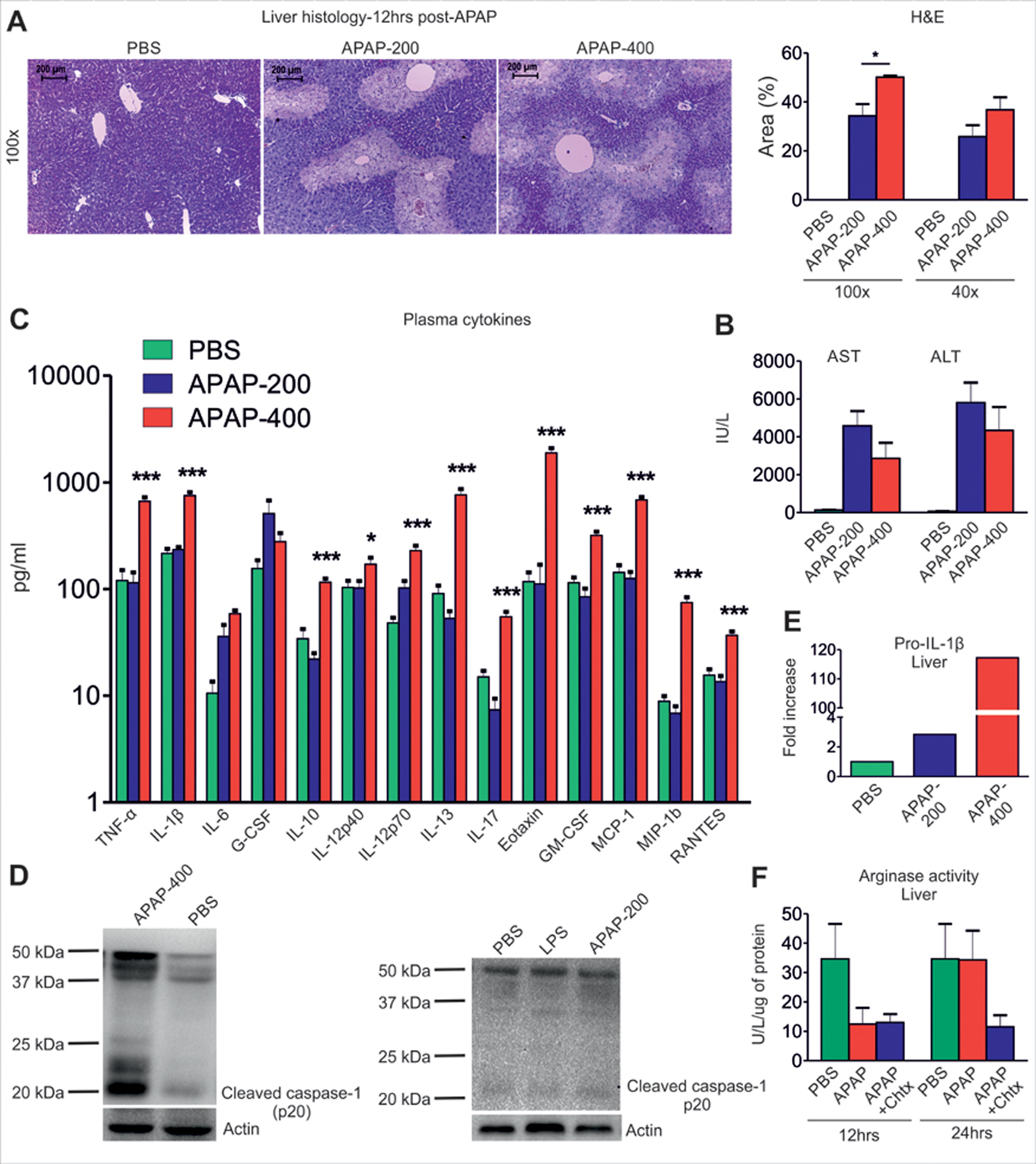

Supplement: Supplementary Figure 1 [file cddis2016131x2.tif]

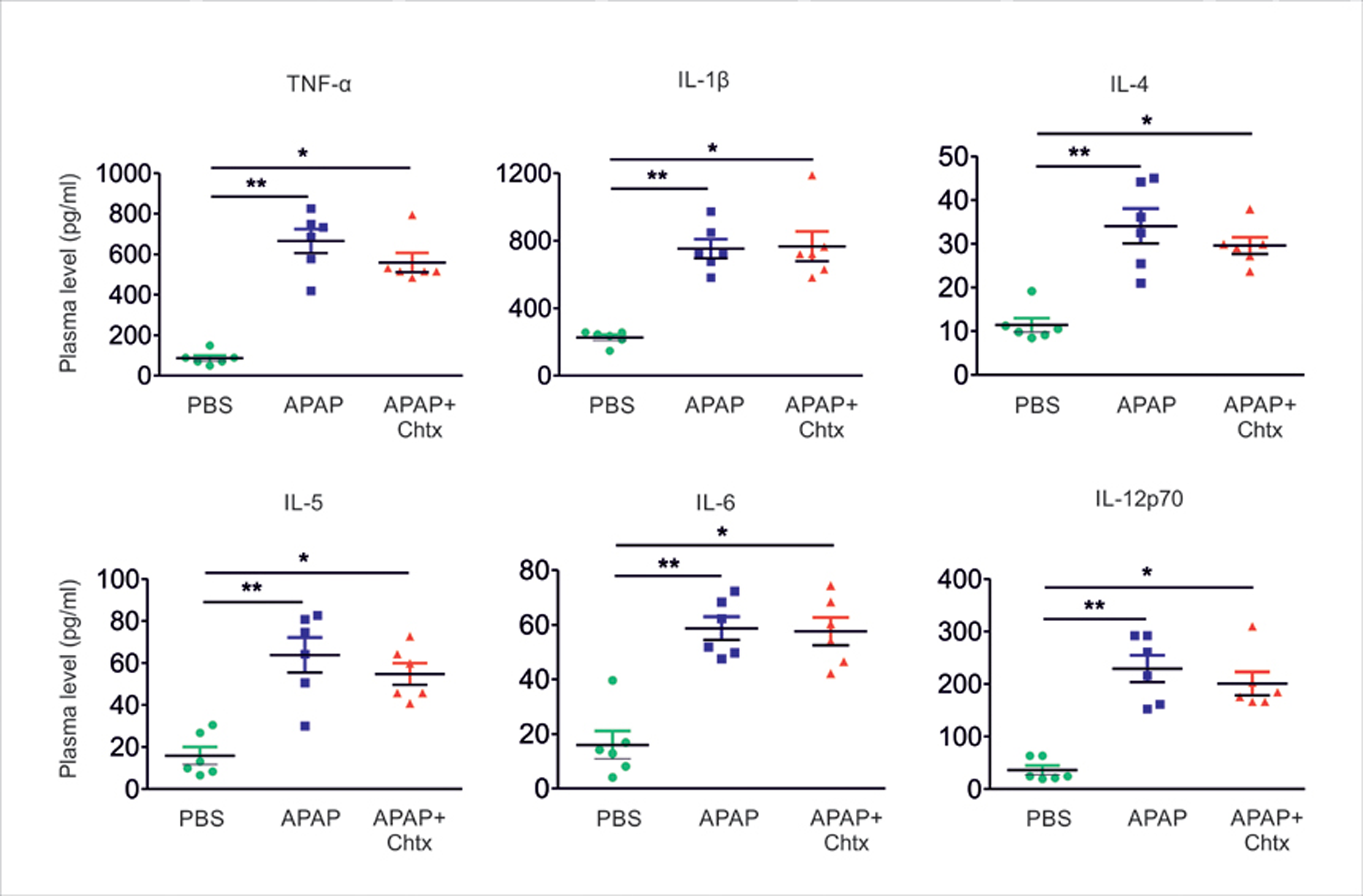

Supplement: Supplementary Figure 2 [file cddis2016131x3.tif]
